# Prevalence of depression and its association with health-related quality of life in people with heart failure in low- and middle-income countries: A systematic review and meta-analysis

**DOI:** 10.1371/journal.pone.0283146

**Published:** 2023-03-23

**Authors:** Henok Mulugeta, Peter M. Sinclair, Amanda Wilson

**Affiliations:** 1 Department of Nursing, College of Health Science, Debre Markos University, Debre Markos, Amhara Region, Ethiopia; 2 School of Nursing and Midwifery, Faculty of Health, University of Technology Sydney, Sydney, New South Wales, Australia; University of Adelaide School of Medical Sciences: The University of Adelaide Adelaide Medical School, AUSTRALIA

## Abstract

**Introduction:**

Heart failure is a growing public health concern around the world. People with heart failure have a high symptom burden, such as depression, which affects health-related quality of life (HRQoL). The objective of this systematic review and meta-analysis was to estimate the pooled prevalence of depression and evaluate its association with HRQoL among people with heart failure in low- and middle-income countries (LMICs).

**Methods:**

This systematic review was conducted in accordance with the JBI methodology. Electronic databases such as MEDLINE, PsycINFO, EMBASE, CINAHL, Web of Science, Scopus and JBI EBP were searched to identify relevant studies published from January 2012 to August 2022. The methodological quality of each article was assessed using relevant JBI critical appraisal instruments. A random-effects model was employed to estimate the pooled prevalence of depression. Heterogeneity across the studies was investigated using Cochrane’s Q test and I^2^ statistic. The Preferred Reporting Items for Systematic Reviews and Meta-analyses guidelines 2020 were followed for reporting the results. All statistical analyses were performed using STATA version 17 software.

**Results:**

After screening, a total of 21 eligible articles with 5074 participants with heart failure were included in this review. The pooled prevalence of depression among people with heart failure in LMICs was 51.5% (95% CI = 39.7, 63.3%, I^2^ = 99.00%). Subgroup analysis revealed, the highest prevalence in studies whose participants were in-patients, and from the Middle East and North Africa, and studies utilizing Becks Depression Inventory (BDI). Depression was positively associated with HRQoL.

**Conclusion:**

This review revealed that almost half of all people with heart failure in low- and middle-income countries have comorbid depression. People with heart failure and depressive symptoms had poor HRQoL. Therefore, early screening of depression is critical for improving HRQoL in this population.

**Systematic review registration:** PROSPERO CRD42022361759.

## Introduction

Cardiovascular diseases (CVD) are the leading cause of mortality globally with an estimated 17.9 million deaths in 2019, accounting for 32% of all deaths [1]. It is predicted that over 23 million people will die annually from CVDs worldwide by 2030 [2]. The burden of CVD is increasing in low- and middle-income countries (LMICs) where 75% of all deaths are related to CVD [3]. This burden can be attributed to a lack of primary health care services to support the early detection and management of cardiovascular risk factors [4].

Heart failure is a major CVD associated with high morbidity and mortality [5]. The global prevalence of heart failure (HF) is increasing due to ageing and population growth, with an estimated 64 million people affected [6, 7]. It is responsible for more than 300,000 global deaths annually [8, 9]. Although there are limited data from population-based studies, the available data from hospital-based studies show that heart failure is increasingly prevalent in low- and middle-income countries (LMICs) [10]. People with heart failure have many debilitating symptoms such as depression and poor health-related quality of life (HRQoL) compared to the general population due to the unpredictable nature of the disease [11, 12].

The psychological impact of HF, such as depression, is increasing significantly and leads to a poor prognosis [13]. People with HF who are depressed have an increased risk of poor HRQoL compared to those without depression [13, 14]. The findings from two recent systematic reviews found the prevalence of any severity of depression in people with heart failure was 42% [15], and the overall HRQoL in these populations was moderate [16]. However, these reviews only included a small number of studies from LMICs. This means there is considerable uncertainty about the prevalence of depression in this region. A systematic review and meta-analysis conducted in China found that 43% of people with heart failure have depressive symptoms [17]. However, this figure does not represent the burden of the problem in LMICs as all data were from China.

While there are many studies on depression and its association with HRQoL among HF patients in LMICs, the results are inconsistent and inconclusive, meaning the current burden of the problem remains unknown in these populations [18]. In this systematic review, we aimed to estimate the regional burden of depression and assess the association between depression and HRQoL in people with HF in LMICs. The findings of this review will provide contemporary evidence with the potential to assist healthcare policymakers and researchers in developing intervention programs and guidelines for improving the management and care of people with heart failure in LMICs.

### Review questions

This review sought to answer the following two questions:

1. What is the prevalence of depression in people with heart failure in LMICs?
2. Is there an association between depression and HRQoL in people with heart failure in LMICs?

## Inclusion and exclusion criteria

### Participants (population)

This review included studies from LMICs whose participants who are 18 years or older and had a confirmed diagnosis of heart failure.

### Condition

The prevalence of depression and/or association of depression with HRQoL in the participants. For the purpose of this review, heart failure is defined as the inability of the heart to effectively pump blood as evidenced by either signs and symptoms based on Framingham criteria or reduced ejection fraction (<40%) [19, 20]. Depression is defined as the persistent feeling of unhappiness and lack of interest in daily activities with symptoms for at least two weeks, based on DSM-5 diagnostic criteria [21]. Health-related quality of life was defined as self-reported physical, mental, emotional, and social health functioning [22].

### Context

#### Low-and-middle income countries

For the purposes of this review, low to middle income countries were defined using the World Bank atlas method [23] based on the stratification of economies based on gross national income (GNI) per capita. Low-income countries are those with a GNI per capita of $US1,045 or less; lower and upper middle-income economies are those with a GNI per capita between $US1,046 and $US4,095 and $US4,096 and $US12,695 and respectively.

### Outcomes

The primary outcome of this review was the prevalence of depression. The secondary outcome was the association between depression and HRQoL scores measured using a psychometrically validated instrument.

#### Types of studies

Observational (cross-sectional, cohort, case-control) studies that reported the prevalence of depression and/or association of depression with HRQoL in people with heart failure.

For the secondary objective of this review, the following inclusion criteria were considered using the PEO (P = Population, E = exposure, O = outcome) model.

#### Population

Adults with a confirmed diagnosis of heart failure.

#### Exposure of interest

Depression.

#### Outcome

HRQoL.

## Methods

### Design

This systematic literature review has followed methodology guidelines outlined by the Joanna Briggs Institute (JBI) methodology for Systematic Reviews [24] and is reported in line with the PRISMA 2020 guidelines [25]. The protocol for this systematic review was registered in the PROSPERO online database (registration number CRD42022361759) and previously published [26].

### Search strategy

The search strategy aimed to locate both published and unpublished studies. Information sources were electronic databases, conference proceedings, websites, dissertations, and direct contact with the author if required. A preliminary original search of MEDLINE (Ovid) and CINAHL (EBSCO) was undertaken in May 2022 and was updated in August 2022. The last search was done on August 20, 2022. The text words in the titles and abstracts of relevant articles and the index terms used to describe the articles were analysed and used to inform a full search strategy in collaboration with a faculty librarian. The search strategy was developed using the **CoCoPop** (**Co** = Condition, **Co** = Context, **Pop** = Population) model considering the **PEO** (P = Population, E = exposure, O = outcome) model for the second research question of this review. The databases searched includes MEDLINE (Ovid), PsycINFO (EBSCOhost), EMBASE (Ovid), CINAHL (EBSCOhost), Web of Science (Direct access), Scopus (Direct access) and JBI EBP database (Ovid). Index terms (subject headings) and keywords used for the search strategy were adapted for each database. The full search strategy for each database is attached in S1 Table. The reference lists of all identified relevant studies and systematic reviews were screened to identify additional studies. A search for unpublished studies was conducted using Google scholar, Mednar, ProQuest and dissertation databases. Articles published in English language from January 2012 to August 2022 were included to establish the most recent estimate.

### Study selection and outcome

Following the search, all identified citations were collated and uploaded into EndNote V20 (Clarivate Analytics, PA, USA). After removing duplicates, two researchers (HM and PS) screened all titles and abstracts from the original search against the predefined inclusion criteria. The full text of selected citations was assessed in detail against the inclusion criteria independently by the reviewers (HM and PS). The reasons for excluding papers were recorded and reported. Any disagreements between the reviewers were resolved through discussion. The search results and the study inclusion process were reported in accordance with the Preferred Reporting Items for Systematic Reviews and Meta-analyses (PRISMA) guidelines [25].

### Quality appraisal

Two independent reviewers (HM and PS) critically appraised the eligible studies for methodological quality using a standardised JBI critical appraisal instrument for studies reporting prevalence data [27]. The tool is comprised of 9 items that focus on target population, sample size adequacy, study subject and setting (context), reliability of condition measurement, appropriateness of the statistical test used to analyse the data, and adequacy of the response rate with the option to answer ‘No’, ‘Yes’, or ‘unclear’. Authors of papers were contacted to request missing or additional data for clarification, where required. Following the critical appraisal, the reviewers included or excluded studies based on the overall appraisal quality. A study was excluded if it had more than three ‘No’ or ‘unclear’ quality categories. This threshold criterion is consistent with that used in a similar published systematic review [28]. The quality of eligible articles to assess the association between depression and HRQoL were also appraised using the JBI cross-sectional studies critical appraisal tool for studies reporting association (etiology/risk) [29]. Any disagreements were resolved through discussion. The results of the critical appraisal are reported in narrative and table form (Tables 1 and 5).

**Table 1:** Methodological quality of included prevalence studies.

Included articles	Q1	Q2	Q3	Q4	Q5	Q6	Q7	Q8	Q9	Quality score/9
Edmealem A. et al. [37]	Y	Y	U	Y	Y	U	Y	Y	Y	7
DeWolfe A, et al. [38]	Y	Y	Y	Y	Y	Y	Y	Y	U	7
Okviansanti F, et al. [39]	U	Y	Y	Y	Y	Y	N	Y	Y	7
Pushkarev GS, et al. [40]	U	Y	Y	Y	Y	Y	U	Y	Y	7
Fan X, et al. [32]	Y	Y	Y	Y	Y	Y	U	Y	Y	8
Zahid I, et al. [41]	U	U	Y	Y	Y	Y	U	Y	Y	7
Yazew KG, et al. [33]	Y	Y	Y	Y	Y	Y	U	Y	Y	8
AbuRuz ME [34]	U	Y	Y	Y	Y	Y	Y	Y	Y	8
Pan S, et al. [42]	U	Y	Y	Y	Y	Y	U	Y	Y	7
Husain MI, et al. [35]	Y	Y	Y	Y	Y	Y	U	Y	Y	8
Tran NN, et al. [36]	U	Y	Y	Y	Y	Y	Y	Y	Y	8
Tsabedze N, et al. [43]	U	Y	Y	Y	Y	Y	U	Y	Y	7
Erceg P, et al. [44]	U	Y	Y	Y	Y	Y	N	Y	Y	7
Alemoush RA, et al. [31]	Y	Y	Y	Y	Y	Y	Y	Y	Y	9
Saima D, et al. [45]	U	Y	Y	Y	Y	Y	U	Y	Y	7
Ghanbari A, et al. [46]	U	Y	Y	Y	Y	Y	U	Y	Y	7
Zhang X, et al. [47]	U	Y	Y	Y	Y	Y	U	Y	Y	7
Khan S, et al. [48]	U	Y	Y	Y	Y	Y	N	Y	Y	7
Molavynejad S, et al. [49]	U	Y	Y	Y	Y	Y	N	Y	Y	7
Son YJ, et al. [50]	U	Y	Y	Y	Y	Y	U	Y	Y	7
Son YJ, et al. [51]	U	Y	Y	Y	Y	Y	U	Y	Y	7

Y = yes; N = No; U = Unclear.

### Data extraction

The JBI data extraction tool for prevalence data and association (etiology/risk) studies were used to extract the following information from each included study for each research question: authors, year of publication, country, region, design, population, sample size, sampling methods, outcome measuring tool, prevalence of depression, HRQoL mean score based on exposure (depression), measure of association, and quality appraisal score. When there was missing data, authors were contacted for relevant information. Two reviewers (HM and AW) independently conducted the primary data extraction and cross-checked for inconsistencies. Any disagreements and discrepancies between the reviewers were resolved by discussion.

### Data analysis and synthesis

A narrative presentation of the outcomes including text, table, and figure, were used to discuss the characteristics of the included studies and synthesise the prevalence of depression and its association with HRQoL. A random-effects model with DerSimonian and Laird model was used to estimate the pooled effect size of depression, as recommended by Tufanaru et al. [30]. Subgroup analyses were conducted to investigate the variation between different study characteristics, such as region, type of outcome measuring instrument, and type of study population. Heterogeneity was assessed statistically using the standard chi-squared and I-squared tests. The sources of heterogeneity were analysed using subgroup analysis, and meta-regression. The presence of publication bias was assessed visually using a funnel plot, and statistical tests for funnel plot asymmetry was checked by Egger test statistics. A leave-one-out sensitivity analysis was also conducted for assessing the influence of each study on the overall effect size estimate. The pooled effect size was presented using a forest plot. All statistical analysis was performed using STATA Version 17 statistical software.

## Results

### Search results

**Fig 1:**
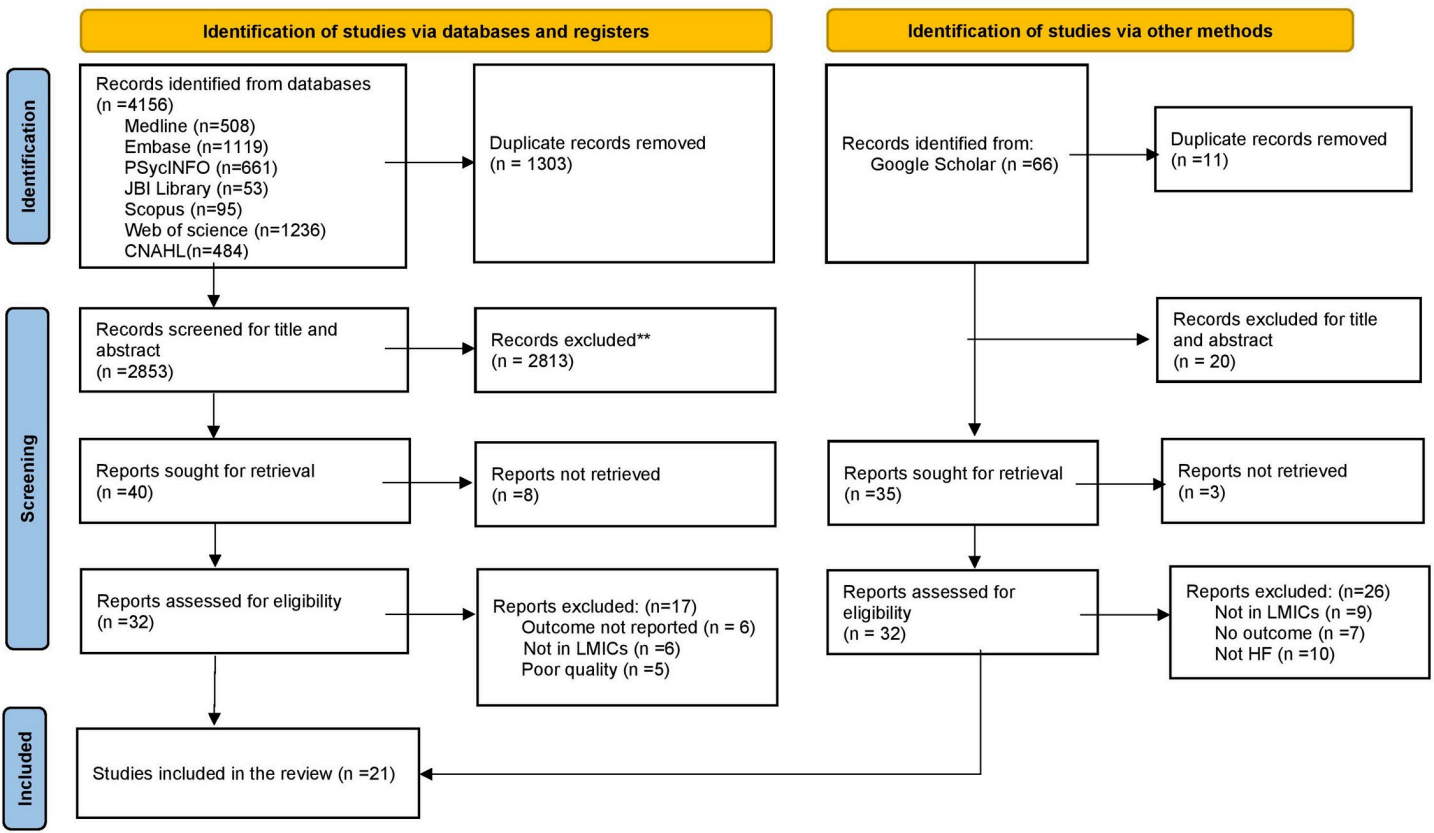
PRISMA flow diagram of literature identification, study selection and inclusion process.

The online electronic search process yielded 4222 articles (4156 from databases and 66 from other sources) of which 1303 were duplicates. After reviewing the title and abstract, we excluded 2844 irrelevant articles. From the remaining 75 articles, 49 were removed after full text assessment. A further five articles were excluded due to poor methodological quality leaving 21 relevant primary research articles eligible for this systematic review (Fig 1).

### Assessment of methodological quality for prevalence studies

This review included 21 articles with moderate to high methodological quality. One study [31] scored 9 points, five studies [32–36] scored 8 points, and the remaining 15 studies [37–51] scored 7 points in the JBI critical appraisal checklist [27] for studies reporting prevalence data (Table 1).

### Overall study characteristics

Of the 21 studies, seven were conducted in East Asia and Pacific region [32, 36, 39, 42, 47, 50, 51], four in the Middle East and North Africa [31, 34, 46, 49], three in Sub-Saharan Africa [33, 37, 43], three in Europe and Central Asia [38, 40, 44], and four in South Asia [35, 41, 45, 48]. Most studies used a descriptive cross-sectional design (n = 17, 81%) and the remaining (n = 4, 19%) were prospective cohort studies. Many (57%) of the studies were conducted in outpatient population, and most (33%) used consecutive sampling technique. The sample size of the included studies ranged from 43 [37] to 1009 [35]. Included studies assessed the prevalence of depression using nine different psychometrically validated instruments. Five studies [32, 35, 40, 45, 49] used Beck Depression Inventory (BDI), five [33, 38, 39, 41, 50] used Patient Health Questionnaire-9 (PHQ-9), four [31, 34, 47, 48] used Hospital Anxiety and Depression Score (HADS), two [44, 51] Geriatric Depression Scale (GDS), and the remaining five [36, 37, 42, 43, 46] each used Cardiac Depression Scale (CDS), Patient Health Questionnaire-2 (PHQ-2), Hamilton Rating Scale for Depression (24-items) (HAM-D_24_), Geriatric Depression Scale (GDS), International Statistical Classification of Diseases and Related Health Problems V10 (ICD-10) (Table 2).

**Table 2:** Characteristics of included studies for prevalence of depression among people with heart failure in LMICs.

ID	Author (reference)	Publication year	Country	Region	Study design	Population	Sample size	Sampling method	Outcome measuring tool	Prevalence	Quality score
1	Edmealem A. et al. [37]	2020	Ethiopia	Sub-Saharan Africa	Cross-Sectional	Outpatient	43	Stratified	PHQ-2	11.1	7
2	DeWolfe A, et al. [38]	2012	Georgia	Europe and Central Asia	Prospective cohort	Outpatient	314	Unreported	PHQ-9	13.0	8
3	Okviansanti F, et al. [39]	2020	Indonesia	East Asia and Pacific	Cross-Sectional	Outpatient	155	Consecutive	PHQ-9	85.2	7
4	Pushkarev GS, et al. [40]	2018	Russia	Europe and central Asia	Prospective cohort	Outpatient	260	Unreported	BDI	60.0	7
5	Fan X, et al. [32]	2015	China	East Asia and Pacific	Cross-Sectional	Inpatient	152	Consecutive	BDI	44.0	8
6	Zahid I, et al. [41]	2018	Pakistan	South Asia	Cross-Sectional	Outpatient	170	Consecutive	PHQ-9	60.0	7
7	Yazew KG, et al. [33]	2019	Ethiopia	Sub-Saharan Africa	Cross-Sectional	Outpatient	422	Systematic random	PHQ-9	51.1	8
8	AbuRuz ME [34]	2018	Jordan	Middle East and North Africa	Cross-Sectional	Outpatient	200	Convenient	HADS	65.0	8
9	Pan S, et al. [42]	2016	China	East Asia and Pacific	Cross-Sectional	Inpatient	366	Consecutive	HAM-D24	57.4	7
10	Husain MI, et al. [35]	2019	Pakistan	South Asia	Cross-Sectional	Outpatient	1009	Unreported	BDI	66.0	8
11	Tran NN, et al. [36]	2022	Vietnam	East Asia and Pacific	Cross-Sectional	Inpatient	128	Convenient	ICD-10	46.9	8
12	Tsabedze N, et al. [43]	2021	South Africa	Sub-Saharan Africa	Cross-Sectional	Outpatient	103	Consecutive	DASS-21	52.4	7
13	Erceg P, et al. [44]	2013	Serbia	Europe and Central Asia	Cross-Sectional	Inpatient	136	Consecutive	GDS	55.9	7
14	Alemoush RA, et al. [31]	2021	Jordan	Middle East and North Africa	Prospective follow up	Outpatient	127	Consecutive	HADS	47.3	9
15	Dastgeer S, et al. [45]	2016	Pakistan	South Asia	Prospective follow up	Inpatient	400	Unreported	BDI	64.7	7
16	Ghanbari A, et al. [46]	2015	Iran	Middle East and North Africa	Cross-Sectional	Inpatient	239	Gradual sampling	CDS	57.7	7
17	Zhang X, et al. [47]	2020	China	East Asia and Pacific	Cross-Sectional	Inpatient	254	Convenient	HADS	18.1	7
18	Khan S, et al. [48]	2012	Pakistan	South Asia	Cross-Sectional	Inpatient	121	Consecutive	HADS	30.0	7
19	Molavynejad S, et al. [49]	2019	Iran	Middle East and North Africa	Cross-Sectional	Inpatient	151	Convenient	BDI	97.0	7
20	Son YJ, et al. [50]	2018	South Korea	East Asia and Pacific	Cross-Sectional	Outpatient	190	Convenient	PHQ-9	30.0	7
21	Son YJ, et al. [51]	2012	South Korea	East Asia and Pacific	Cross-Sectional	Outpatient	134	Unreported	GDS	67.9	7

### Prevalence of depression in people with heart failure in LMICs

**Fig 2:**
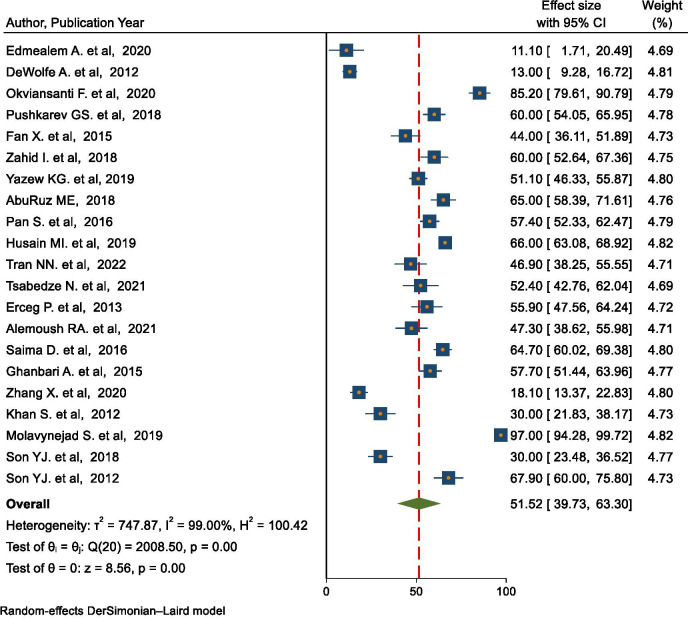
The pooled prevalence of depression in people with heart failure in LMICs.

In total, 21 studies reported the prevalence of depression in people with heart failure in LMICs. The lowest and the highest prevalence of depression were 11.1% [37] and 97.0% [49], respectively (Table 1). The pooled regional prevalence of depression among people with heart failure in LMICs was 52% (95% CI = 39.73, 63.3%, I^2^ = 99.00%). The overall pooled effect size of depression presented using forest plot (Fig 2).

### Sub-group analysis

Subgroup analysis was done using region where the studies were conducted, study population and the outcome measuring instrument. The result showed that the highest prevalence was observed among studies conducted in Middle East and North Africa, among inpatients and studies that screened depression using BDI (Table 3).

**Table 3:** Subgroup analysis on the prevalence of depression among people with heart failure in LMICs.

Subgroup	Number of studies	Sample size	Pooled Prevalence	Heterogeneity	
				*I* ^*2*^	P-value
By region					
East Asia and pacific	7	1379	49.91	98.5	<0.001
South Asia	4	3708	55.62	95.6	<0.001
Middle East and North Africa	4	717	66.91	98.8	<0.001
Sub -Saharan Africa	3	568	38.35	96.6	<0.001
Europe and Central Asia	3	710	42.86	99.1	<0.001
Latin America and Caribbean	0	…	….	…	…
By Population					
Outpatient	12	3127	50.79	98.6	<0.001
Inpatient	9	1947	52.47	99.2	<0.001
By outcome measurement tool					
BDI	5	1972	66.51	99	<0.001
PHQ-9	5	1251	47.82	99.2	<0.001
HADS	4	702	40.04	97.8	<0.001
GDS	2	270	61.98	76.1	0.04
Others (CDS, HAM-D24, PHQ-2, ICD-10, DASS-21)	5	879	45.35	95	<0.001

### Assessment of heterogeneity

The result of this meta-analysis using the random-effects model revealed a high heterogeneity across the included studies (I^2^ = 99%, P = 0.001). Heterogeneity is inevitable in meta-analysis due to difference in study quality, methodology, sample size and inclusion criteria for participants [52, 53]. Consequently**,** meta-regression was performed using publication year, sample size and quality score as covariates to find possible sources of heterogeneity among the included studies. The result of the meta-regression analysis showed that no significant linear relationship existed between the outcome (depression) and the covariates. Therefore, none of the three covariates were significantly associated with the presence of heterogeneity (Table 4). The high heterogeneity can be attributed to chance or other factors not included in this review.

**Table 4:** Meta-regression analysis of factors associated with heterogeneity.

Heterogeneity source	Coefficients	Standard Error	P-value
Sample size	0.02	0.03	0.49
Publication Year	1.09	1.99	0.58
Quality score	-7.53	10.90	0.49

### Assessment of publication bias

**Fig 3:**
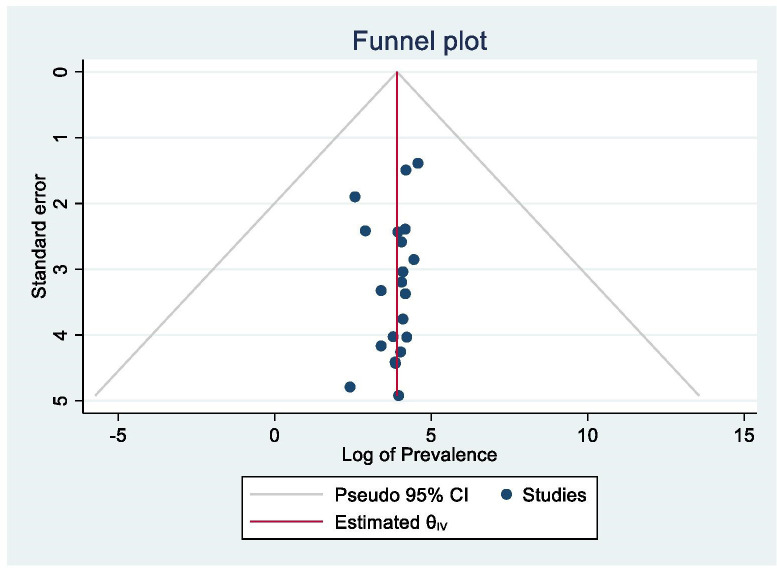
Funnel plot to test the publication bias of the 21 studies.

Visual inspection of the funnel plot suggested asymmetry, as eight studies lay to the left and 13 to the right of the line (Fig 3). However, this was not statistically significant as evidenced by Egger’s test (P = 0.81), which confirmed the results were not influenced by publication bias. It is worth noting that asymmetry in funnel plots is not always linked to publications bias [54], and that high heterogeneity may explain the visual asymmetry in the funnel plot.

#### Sensitivity analysis

**Fig 4:**
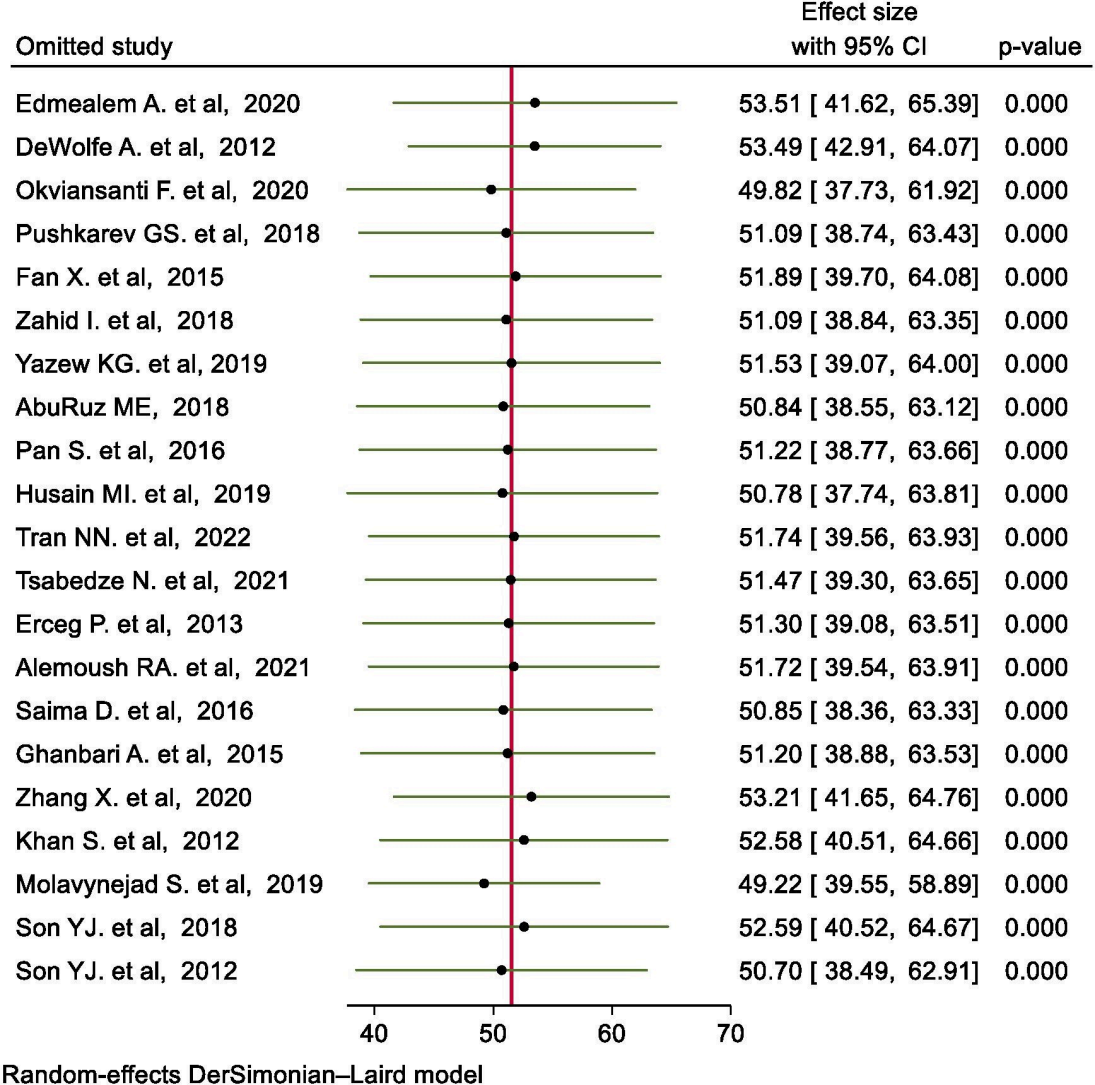
Result of sensitivity analysis of the 21 studies.

The result of leave-one-out sensitivity analysis using a random effects model demonstrated that no single study unduly influenced the pooled estimate of depression. For each study, the displayed effect size corresponds with the overall effect size computed from meta-analysis excluding that study (Fig 4).

### The association between depression and HRQoL

Of the 21 eligible studies, only six reported an association between depression and HRQoL using depression as exposure variable and HRQoL as outcome. There were variations in the ways that depression and HRQoL were measured among these studies. For instance, two studies [44, 51] used GDS, two studies [31, 34] used HADS, one study [43] used DASS-21 and one study [38] used PHQ-9 to measure depression. Likewise, four studies [38, 43, 44, 51] used MLHFQ and the other two studies [31, 34] used SF-36 to measure HRQoL. Regarding the critical appraisal, the JBI cross-sectional studies critical appraisal tool for studies reporting association (etiology/risk) was used to assess the quality of each study, and the result showed that all six studies had good methodological quality (Table 5).

**Table 5:** Methodological quality of included studies for association of depression and HRQoL.

Included articles	Q1	Q2	Q3	Q4	Q5	Q6	Q7	Q8	Quality score/8
DeWolfe A, et al. [38]	Y	Y	Y	Y	Y	Y	Y	Y	8
AbuRuz ME [34]	Y	Y	Y	Y	Y	Y	Y	Y	8
Tsabedze N, et al. [43]	Y	Y	Y	Y	Y	Y	Y	Y	8
Erceg P, et al. [44]	Y	Y	Y	Y	Y	Y	Y	Y	8
Alemoush RA, et al. [31]	Y	Y	Y	Y	Y	Y	Y	Y	8
Son YJ, et al. [50]	Y	Y	Y	Y	Y	Y	Y	Y	8

Y = yes; N = No; U = Unclear.

Concerning the effect size, five studies [31, 34, 38, 44, 51] used beta(β) as effect size to report the association between depression and HRQoL, while one study [43] used Odds Ratio (OR) to indicate the strength of association, with all six studies reporting a statistically significant association between depression and HRQoL. All included studies evaluating the association between depression as the exposure variable and HRQoL as an outcome are profiled in Table 6.

**Table 6:** The association between depression and health-related quality of life in people with heart failure.

Author [year of publication]	Country	Sample size	Depression measuring tool	HRQoL measuring tool	Outcome (HRQoL score) based on exposure		Type of comparison	Outcome measure with result	Conclusion
					Depressed	Non-Depressed			
Erceg P, et al. [2013] [44]	Serbia	136	GDS	MLHFQ	57.9±17.6	40.9±17.1	Linear regression	β = 0.41, P = 0.001	1 unit increase in the depression score was associated with a 0.41 unit increase in MLHFQ QoL score
Tsabedze N, et al. [2021] [43]	South Africa	103	DASS-21	MLHFQ	28 (10–54)	5(0–17)	Logistic regression	OR = 1.04, P = 0.001	Depressed people are 1.04 times more likely to have poor HRQoL as compared to non-depressed one
Son YJ, et al. [2012] [51]	South Korea	134	GDS	MLHFQ	59.4±9.97	45.1±8.8	Linear regression	β = 0.44, P = 0.001	1 unit increase in the depression score was associated with a 0.44 unit increase in MLHFQ QoL score
DeWolfe A, et al. [2012] [38]	Georgia	314	PHQ-9	MLHFQ,	74.9±11.9	58.4±13.5	Linear regression	β = 1.83, P = 0.001	1 unit increase in the depression score was associated with a 1.83 unit increase in MLHFQ QoL score
Alemoush RA, et al. [2021] [31]	Jordan	127	HADS	SF-36	……	………	Linear regression	β = -0.37, P = 0.001	1 unit increase in the depression score was associated with a 0.37 unit decrease in SF-36 QoL score
AbuRuz ME [2018] [34]	Jordan	200	HADS	SF-36	……	………	Linear regression	β = -0.32, P = 0.001	1 unit increase in the depression score was associated with a 0.32 unit decrease in SF-36 QoL score

## Discussion

The burden of heart failure has increased over the past decade in LMICs with a significant economic impact and alteration in psychological, physical, and emotional well-being [55]. Evidence regarding the burden of depression and its impact on HRQoL in people with HF from LMICs is limited. This review was conducted to estimate the pooled regional prevalence of depression, and to investigate the association between depression and HRQoL among people with heart failure in LMICs. To our knowledge, this is the first review to estimate the current prevalence of depression in people with HF in LMICs. The result of this review revealed that the pooled regional prevalence of depression in people with heart failure in LMICs was 51.5% (95% CI = 39.73, 63.30%, I^2^ = 99.00%). This reinforces the understanding that depression is a common comorbid condition in people with heart failure and is consistent with the findings of the previous systematic review [56]. Our estimate is higher than the global prevalence of depression in people with heart failure [15]. The higher prevalence in LMICs might be due to variation in the socio-demographic characteristics of the study participants, discrepancy of instruments, sample size, study setting, and level of economic status [35, 57].

The subgroup analysis of this review showed significant variation in the prevalence of depression among different groups. For instance, the highest (66.9%) and the lowest (38.4%) pooled prevalence was observed in studies from the Middle East and North Africa regions and Europe and Central Asia, respectively. This variation might be due to socioeconomical, health care coverage, sample size and methodological differences among the included studies across the regions. In the present review, the prevalence of depression is higher among inpatients than outpatients. A similar finding was observed in the previous systematic review [58]. This might be due to the severity of the disease or the fact that hospitalized patients are more unwell and have more socioeconomic burdens than outpatients. Consistent with the previous systematic review conducted in China [17], the pooled prevalence of depression in the current review was highest (66.5%) when measured using Beck Depression Inventory (BDI). The lowest prevalence of depression (40.1%) was observed when measured using Hospital Anxiety and Depression Score (HADS). This difference could be due to differences in definitions of depression and cut-off points to diagnose depression across the various scales. However, further research would be helpful to investigate the factors that might lead to such differences across the depression measuring scales.

The association of depression with HRQoL has been reported in several recent studies. The results of this systematic review also demonstrated that six studies among the included studies showed a significant association between depression and HRQoL, although there were insufficient data to estimate the pooled effect size. This finding is similar to previous studies conducted in Europe [59–61]. These studies found that people with heart failure who have depressive symptoms had poor quality of life compared to those who did not have depressive symptoms, and this was also correlated with an increased burden of morbidity and mortality due to HF [51]. The findings of this review highlight the need to understand the factors that contribute to the increased incidence of depression in people with heart failure living in LMICs, as well as the factors that contribute to a poorer quality of life. This will enable targeted interventions and support strategies to be designed and evaluated to improve outcomes for this population.

The findings of this meta-analysis have implications for clinical practice. We included articles published between 2012 and 2022. This cut-off date was decided arbitrarily by the authors to estimate the most recent prevalence rate which should have more relevance to current clinical practice. Determining the most recent prevalence of depression provides up-to-date evidence to develop comorbid depression prevention strategies in this group and ultimately improving HRQoL. Determining the effect of depression on HRQoL can help health-care providers prioritize during their routine clinical practice, which will reduce the overall burden of morbidity and mortality. However, some limitations should be considered for future research. First, the interpretation of the results must be taken cautiously as the meta-analysis had statistically significant heterogeneity across the included studies which was not fully explained by the variables examined. Second, the conclusion of positive association between depression and HRQoL, as reported in six studies, should be interpreted cautiously due to our inability to summarise the pooled effect size.

## Conclusion

This systematic review and meta-analysis revealed that one in two people with heart failure in LMICs have comorbid depression. Depression was positively associated with HRQoL in people with heart failure. Early detection and treatment of depression in people with heart failure is highly recommended to reduce its burden in LMICs. Future research should investigate the factors associated with depression and HRQoL in this population.

## Supporting information

S1 Table Search strategy of databases from January 2012 to August 2022. (DOCX)

S1 Checklist PRISMA 2020 checklist. (DOCX)

## List of abbreviations

CVD: Cardiovascular diseases
HF: Heart failure
HRQoL: Health-related quality of life
JBI: Joanna Briggs Institute
LMICs: Low and middle income countries
PRISMA: Preferred Reporting Items for Systematic reviews and Meta-Analyses
