# Supplementary material for: Prevalence of depression and its association with health-related quality of life in people with heart failure in low- and middle-income countries: A systematic review and meta-analysis
Source: PLoS One. 2023 Mar 23;18(3):e0283146. doi: 10.1371/journal.pone.0283146 (PMC10035817; doi:10.1371/journal.pone.0283146)
Supplement: S1 Table — (DOCX) [file pone.0283146.s001.docx]

**S1 Table: Search strategy of databases from January 2012 to August 2022**

| **Medline** | | |
| --- | --- | --- |
| **No** | **Query** | **Records retrieved** |
| 1 | Depression/ or Depression.tw. | 413590 |
| 2 | Quality of life.tw. or "Quality of Life"/ | 391275 |
| 3 | Health-related quality of life.tw. | 51063 |
| 4 | HRQoL.tw. | 19568 |
| 5 | QoL.tw. | 45371 |
| 6 | Heart failure.tw. or Heart Failure/ | 227166 |
| 7 | heart disease*.tw. or heart diseases/ | 236293 |
| 8 | cardiac failure.tw. | 12328 |
| 9 | **#**1 OR **#**2 OR **#**3 OR **#**4 OR **#**5 | 765187 |
| 10 | Low-middle-income countries* | 1809560 |
| 11 | **#**6 OR **#**7 OR **#**8 | 446521 |
| 12 | **#**9 AND **#**10 AND **#**11 | 1230 |
| 13 | limit **#**12 to "all adult (19 plus years)" | 754 |
| 14 | Limit #13 to English, yr="2012 -Current | 508 |
| **Embase** | | |
| **No** | **Query** | **Records retrieved** |
| 1 | Depression/ or Depression.tw. | 695337 |
| 2 | Quality of life.tw. or "Quality of Life"/ | 688384 |
| 3 | Health-related quality of life.tw. | 75596 |
| 4 | HRQoL.tw. | 32656 |
| 5 | QoL.tw. | 87373 |
| 6 | Heart failure.tw. or Heart Failure/ | 406054 |
| 7 | heart disease*.tw. or heart diseases/ | 336758 |
| 8 | cardiac failure.tw. | 17910 |
| 9 | **#**1 OR **#**2 OR **#**3 OR **#**4 OR **#**5 | 1308642 |
| 10 | Low-middle-income countries* | 2111652 |
| 11 | **#**6 OR **#**7 OR **#**8 | 704966 |
| 12 | **#**9 AND **#**10 AND **#**11 | 2544 |
| 13 | limit **#**12 to (adult <18 to 64 years> OR aged <65+ years>) | 1578 |
| 14 | limit **#**13 to English, yr="2012 -Current" | 1119 |
| **JBI database** | | |
| **No** | **Query** | **Records retrieved** |
| 1 | Depression.mp. | 1323 |
| 2 | Health-related quality of life.mp. | 381 |
| 3 | HRQoL.mp. | 75 |
| 4 | quality of life.mp. | 1816 |
| 5 | QoL.mp. | 221 |
| 6 | Heart failure.mp. | 310 |
| 7 | heart disease*.mp. | 377 |
| 8 | cardiac failure.mp. | 55 |
| 9 | **#**1 or **#**2 or **#**3 or **#**4 or **#**5 | 2507 |
| 10 | Low-middle-income countries* | 1468 |
| 11 | **#**6 or **#**7 or **#**8 | 593 |
| 12 | **#**9 and **#**10 and **#**11 | 138 |
| 13 | limit **#**12 to English, yr="2012 -Current" | 53 |
| **Web of science** | | |
| **No** | **Query** | **Records retrieved** |
| 1 | **TS=(Depression OR "Quality of life" OR "Health-related quality of life" OR HRQoL OR QoL )** | [1061448](https://www.webofscience.com/wos/woscc/summary/8db14632-fc15-4905-8c46-3ee308097c4b-3a8b946e/relevance/1) |
| 2 | **TS=("Heart failure" OR "heart disease*" OR "cardiac failure" )** | [574654](https://www.webofscience.com/wos/woscc/summary/a84fbfac-3ada-4355-8a6b-f854e86b6c15-3a8b9a0b/relevance/1) |
| 3 | Low-middle-income countries* | [4199317](https://www.webofscience.com/wos/woscc/summary/20b22c51-241f-4b70-88be-7afa9fb5ff73-3a8ba198/relevance/1) |
| 4 | **#1 OR #2** | [1703](https://www.webofscience.com/wos/woscc/summary/a0a3f54c-98b3-49d2-ab31-e34963de6731-3a8ba2c1/relevance/1) |
| 5 | **#3 AND #4** | 1426 |
| 6 | **Limit 5 to English**(Languages), yr="2012 -Current" | 1236 |
| **PSycINFO** | | |
| **No** | **Query** | **Records retrieved** |
| 1 | **DE "Major Depression" OR DE "Depression" OR "Depression"** | 530173 |
| 2 | **DE "Health-related quality of life” OR DE "Health-related quality of life" Quality of life" OR OR HRQoL OR QoL)** | 123199 |
| 3 | **DE “Heart disorders" OR "Heart failure" OR "heart disease*" OR "cardiac failure"** | 26789 |
| 4 | Low-middle-income countries* | 581990 |
| 5 | **#1 OR #2** | 416168 |
| 6 | **#3 AND #4 AND #5** | 863 |
| 7 | Limit #**6 to English**(Languages), yr="2012 -Current" | 661 |
| **Low-middle-income countries***  *=(afghanistan or albania or algeria or american samoa or angola or argentina or armenia or azerbaijan or bangladesh or belarus or belize or benin or bhutan or bolivia or "bosnia and Herzegovina" or botswana or brazil or bulgaria or burkina faso or burundi or cabo verde or cambodia or cameroon or central african republic or chad or china or colombia or comoros or congo or costa rica or "cote d'ivoire" or cuba or djibouti or dominica* or ecuador or egypt or arab republic or el salvador or equatorial guinea or eritrea or eswatini or ethiopia or fiji or gabon or gambia or georgia or ghana or grenada or guatemala or guinea or guinea bissau or guyana or haiti or honduras or india or indonesia or iran or iraq or jamaica or jordan or kazakhstan or kenya or kiribati or korea or kosovo or kyrgyz republic or lebanon or lesotho or liberia or libya or madagascar or malawi or malaysia or maldives or mali or marshall islands or mauritania or mauritius or mexico or micronesia or moldova or mongolia or montenegro or morocco or mozambique or myanmar or namibia or nepal or nicaragua or niger or nigeria or north macedonia or pakistan or panama or papua new guinea or paraguay or peru or philippines or romania or russia* or rwanda or samoa or "sao tome and principe" or senegal or serbia or sierra leone or solomon islands or somalia or south africa or south sudan or sri lanka or "st. lucia" or "st. vincent and the grenadines" or sudan or suriname or syrian arab republic or tajikistan or tanzania or thailand or timor leste or togo or tonga or tunisia or turkey or turkmenistan or tuvalu or uganda or ukraine or uzbekistan or vanuatu or vietnam or west bank or gaza or yemen or zambia or zimbabwe or low income countr* or middle income countr* or developing countr*).ti,ab,sh,kf. | | |
